# Paracoccidioidomycosis in Brazil: 25‐Year Nationwide Trends in Mortality, Hospitalisations and In‐Hospital Deaths of a Neglected Systemic Mycosis

**DOI:** 10.1111/tmi.70135

**Published:** 2026-03-24

**Authors:** Anderson Fuentes Ferreira, Jorg Heukelbach, Eliana Amorim de Souza, Maria Aparecida Shikanai‐Yasuda, Lisandra Serra Damasceno, Terezinha do Menino Jesus Silva Leitão, Ziadir Francisco Coutinho, Helia Kawa, Milena Maria Alves Vasconcelos, Alberto Novaes Ramos

**Affiliations:** ^1^ Programa de Pós‐graduação em Saúde Pública, Faculdade de Medicina Universidade Federal do Ceará Fortaleza Brazil; ^2^ Instituto Multidisciplinar em Saúde Universidade Federal da Bahia, Campus Anísio Teixeira Vitória da Conquista Bahia Brazil; ^3^ Departamento de Infectologia e Medicina Tropical, Faculdade de Medicina Universidade de São Paulo São Paulo Brazil; ^4^ Laboratório de Investigação Médica em Imunologia (LIM 48), Hospital das Clínicas, Faculdade de Medicina Universidade de São Paulo São Paulo Brazil; ^5^ Departamento de Saúde Comunitária, Faculdade de Medicina Universidade Federal do Ceará Fortaleza Ceará Brazil; ^6^ Escola Nacional de Saúde Pública Fundação Oswaldo Cruz Rio de Janeiro Rio de Janeiro Brazil; ^7^ Departamento de Epidemiologia e Bioestatística Universidade Federal Fluminense Niterói Rio de Janeiro Brazil; ^8^ Faculdade de Medicina Universidade Federal do Ceará Fortaleza Ceará Brazil

**Keywords:** hospitalisation, in‐hospital mortality, mortality, neglected tropical diseases, paracoccidioidomycosis, spatio‐temporal analysis

## Abstract

**Objective:**

To analyse mortality, hospitalisations and in‐hospital mortality related to paracoccidioidomycosis (PCM) in Brazil, 2000–2024, from a spatio‐temporal and social inequalities perspective.

**Methods:**

We conducted a mixed ecological study using death certificates from the Mortality Information System and hospital admissions from the Hospital Information System of the Brazilian Unified Health System, including admissions that resulted in death. Records with PCM (ICD‐10 B40–B41) as the underlying or associated cause of death, or as the primary or secondary diagnosis, were included. Sex‐ and age‐standardised rates (per 1,000,000 inhabitants) were estimated for Brazil and macro‐regions. Time trends were assessed using Joinpoint regression and spatial distribution was evaluated by health region.

**Results:**

We identified 4904 deaths mentioning PCM over the 25‐year period (2980 [60.8%] as the underlying cause and 1924 [39.2%] as an associated cause) corresponding to a standardised mortality rate of 1.00/1,000,000 inhabitants. Deaths occurred predominantly among men of working age. Mortality declined nationally (average annual percent change [AAPC] −3.85%). There were 18,239 hospital admissions (standardised hospitalisation rate 3.71/1,000,000), which also decreased over time (AAPC −2.83%). In total, 1136 admissions resulted in death (standardised in‐hospital mortality rate 0.23/1,000,000), with no significant overall trend. The highest mortality, hospitalisation and in‐hospital mortality rates clustered in Rondônia, northern Mato Grosso and health regions in the Southeast and Central‐West.

**Conclusions:**

Despite declining national rates, PCM remains concentrated in specific endemic territories, disproportionately affecting socially vulnerable populations and reinforcing its status as a neglected systemic mycosis in Brazil. Making PCM a nationally notifiable disease is essential to reduce its burden.

## 1 Introduction

Paracoccidioidomycosis (PCM) is a systemic mycosis endemic in the Americas. Molecular biology studies have redefined the aetiological agent as a species complex, including *Paracoccidioides brasiliensis* sensu stricto, *P. americana* , *P. restrepiensis*, *P. venezuelensis* and *P. lutzii* , with distinct geographical distributions across Latin America; *P. lutzii* predominates in the Central‐West and Amazon regions of Brazil [1, 2, 3, 4, 5]. There is evidence that antigenic differences may influence the performance of serological tests, surveillance and understanding of the actual burden of disease [1, 2, 3, 6, 7].

PCM is part of the group of endemic mycoses discussed within the scope of neglected tropical diseases (NTDs), and in 2022, the World Health Organisation included Paracoccidioides spp. in its fungal priority pathogens list [8, 9, 10].

Brazil accounts for most reported PCM cases worldwide and is the leading endemic country [1, 2, 4]. The disease is strongly associated with rural settings, agricultural frontiers and environmental transformation, with infection occurring after exposure to fungal propagules in soil [2, 5, 6, 7].

The chronic form predominates in adult men of working age and is frequently associated with pulmonary, mucocutaneous and other sequelae, disability and premature mortality. The acute/subacute form primarily affects children and young adults and may progress rapidly without early treatment [2, 3, 8].

The expansion of agriculture and cattle raising, deforestation, new roads, large infrastructure projects and land‐use change in the Amazon and Cerrado have been linked to the emergence and re‐emergence of cases, particularly in areas of recent colonisation [11, 12, 13, 14]. Recent studies have shown a concentration of cases in regions of intense agribusiness expansion, reinforcing the role of socioenvironmental processes in the spatio‐temporal reconfiguration of PCM [12, 14, 15, 16].

Despite advances in diagnosis, clinical classification, therapeutic management and follow‐up, there remains marked asymmetry between the knowledge accumulated in clinical series and the understanding of the population burden, mortality patterns and use of hospital services within the Brazilian Unified Health System (*Sistema Único de Saúde*, SUS) [2, 3, 5, 17, 18, 19, 20].

PCM is not currently included on the Brazilian national list of compulsorily notifiable diseases, which limits monitoring of incidence, spatial distribution and case fatality. Surveillance, therefore, remains fragmented and often depends on local initiatives and secondary data sources [21, 22]. Given the increasing occurrence of PCM–HIV co‐infection, which may be associated with severe disease and death, strengthening surveillance is particularly important [23]. Some endemic states in Brazil have implemented specific reporting strategies for PCM surveillance, either through state‐level mandatory notification lists or through structured laboratory and clinical systems, as in Rondônia (since 1999), Paraná (2000), Minas Gerais (2012), Goiás (2013), Mato Grosso states (2015) [11, 12, 24] and are included in the compulsory notification list of the states of Mato Grosso do Sul (Resolution No. 693/SES/MS, of 12 December 2005), São Paulo (Resolution SS No. 88, of 24 April 2024) and Rio de Janeiro (Resolution SES No. 2485 of 18 October 2021). These experiences illustrate that routine reporting can support more robust estimation of incidence, identification of high‐risk areas and better‐informed public policies and care pathways.

In the absence of a continuous national case notification system, secondary health information systems play a strategic role in understanding the endemic situation. Among these, the Mortality Information System (SIM) and the Hospital Information System of the SUS (SIH‐SUS) are particularly relevant [21, 22, 25, 26, 27]. Previous work using SIM and SIH‐SUS revealed substantial PCM‐related mortality and hospital morbidity in earlier periods, especially in the South, Southeast and Central‐West [21, 22].

Comparative analyses of mortality, hospital admissions and in‐hospital mortality using nationwide information systems can therefore help characterise the current burden, reveal spatio‐temporal patterns and inform discussions on surveillance and equity. The incorporation of the Brazilian Deprivation Index (*Índice Brasileiro de Privação*, IBP) and municipal typologies further connects this agenda to territorial inequalities within the SUS.

This study analysed PCM‐related mortality, hospital admissions and in‐hospital mortality in Brazil from 2000 to 2024 from a spatio‐temporal and social‐inequalities perspective, using SIM and SIH‐SUS.

## 2 Methods

### 2.1 Study Design

We conducted a nationwide mixed‐ecological study using population‐level data, focusing on three outcomes related to PCM: overall mortality, hospital admissions and in‐hospital mortality. Time‐trend and spatial distribution analyses were performed for the period 2000–2024.

The analysis interval included both the pre‐COVID‐19 period and the years of most significant impact of the public health emergency (2020–2022), as well as the immediate post‐pandemic period (2023–2024).

### 2.2 Study Setting

**FIGURE 1:**
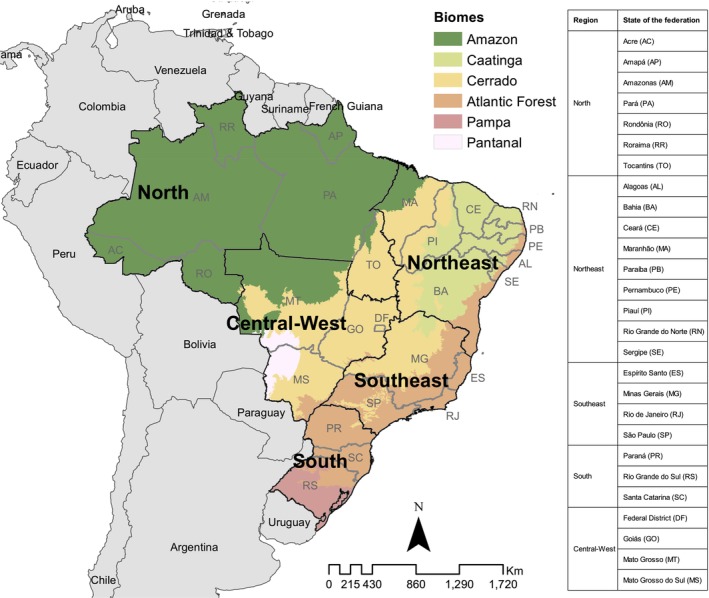
Study area: Brazilian states, macro‐regions and biomes.

The study was conducted in Brazil, a country with an area of approximately 8.5 million km^2^, the most extensive territory in South America, with a population of 203 million inhabitants in 2022 (density of 23.86 inhabitants/km^2^). The political‐administrative organisation comprises 26 states, the Federal District and 5570 municipalities grouped into five macro‐regions (Figure 1) [28]. Spatial analyses used 439 health regions, territorial units adopted for planning regionalised care in the SUS [29, 30].

Beyond the political‐administrative division, the country comprises six major biomes with distinct land‐use patterns. Because PCM is influenced by socioenvironmental change, the interpretation of its distribution considers especially the Amazon and Cerrado (Figure 1) [11, 12, 13, 14, 31].

According to the 2022 Demographic Census, marked inequalities in sanitation and service provision persist in Brazil [28] and hospital resources remain more concentrated in the Southeast and South [29].

### 2.3 Data Sources

Data on deaths and hospital admissions for 2000–2024 were obtained from SIM and SIH‐SUS through DATASUS (Department of Informatics of the SUS) [32]. SIM is based on death certificates, whereas SIH‐SUS compiles hospital admission authorisations from public and contracted SUS facilities. Both systems include individualised records with sociodemographic and clinical information.

Because SIH‐SUS has no unique personal identifier, an individual may have more than one AIH over time; therefore, analyses refer to admissions rather than patients.

PCM‐related records were identified using ICD‐10 code B41 and its subcategories, as underlying or associated causes of death in SIM and as primary or secondary diagnoses in SIH‐SUS. To minimise under‐ascertainment related to legacy terminology, we also included ICD‐10 code B40 (blastomycosis), given the historical use of South American blastomycosis for PCM [21, 33].

Population denominators were obtained from DATASUS, based on the 2000, 2010 and 2022 censuses and intercensal annual estimates, by municipality, sex and age [34].

SIM and SIH‐SUS files were downloaded by state and year, converted from .dbc to .dbf and merged into national databases. Data management, record selection and analyses were performed in Stata 11.2 [35].

### 2.4 Outcomes and Variables

Three main outcomes were defined: PCM mortality, PCM hospital admissions and PCM in‐hospital mortality. Crude and age‐ and sex‐standardised rates were calculated per 1,000,000 inhabitants using the direct method and the 2010 Brazilian population as the standard.

We also identified whether PCM appeared as an underlying or associated cause on death certificates and as a primary or secondary diagnosis in AIH, and we described the most frequent related causes and diagnoses.

Analyses also considered a set of sociodemographic and contextual variables: sex (male, female), age group (0–14, 15–29, 30–39, 40–49, 50–59, 60–69, ≥ 70 years); ethnicity (Caucasian, Afro‐Brazilian, Mixed/Brazilian Pardo, Indigenous, Asian); region of residence (North, Northeast, Southeast, South, Central‐West); municipality population size—Small I (≤ 20,000 inhabitants), Small II (20,001–50,000), Medium (50,001–100,000) and Large (> 100,000 inhabitants); municipality, municipal deprivation according to the IBP (very low, low, medium, high and very high) [36]; municipal typology according to IBGE (urban, intermediate‐adjacent, intermediate‐remote, rural‐adjacent, rural‐remote) [30], place of death (hospital, home, public road, other) for mortality (in SIM) and mention of HIV/AIDS (HIV‐PCM co‐infection) as a basic/associated cause (SIM) or primary/secondary diagnosis (SIH‐SUS) co‐recording in both systems [30, 36, 37].

The IBP was used as a synthetic indicator of material deprivation and socioeconomic position, based on the proportions of households with low per capita income, people with low schooling and families with inadequate sanitation and water supply [36, 37]. The IBGE municipal typology was used to capture differences in urban–rural configuration and access to services [30, 36, 37].

### 2.5 Descriptive Analysis

We described absolute and relative frequencies of the three outcomes and selected variables, including the ‘unknown’ category where applicable.

Crude and age‐ and sex‐standardised rates were estimated for Brazil and macro‐regions.

### 2.6 Time‐Trend Analysis

Time trends of age‐ and sex‐standardised PCM mortality, hospitalisation and in‐hospital mortality rates were assessed using Joinpoint regression [38].

For each segment, annual percent change (APC) and average annual percent change (AAPC) were estimated with 95% confidence intervals. Trends were classified as increasing, decreasing or with no clear overall trend, and significance was assessed using Monte Carlo permutation tests with a 5% significance level. The Joinpoint Regression Program version 5.2.0.0 (National Cancer Institute, USA) was used [38].

### 2.7 Spatial Analyses

Spatial analyses used health regions (*n* = 439) as the unit of aggregation. To reduce instability due to small numbers, rates were estimated for five aggregated periods (2000–2004, 2005–2009, 2010–2014, 2015–2019 and 2020–2024), standardised by age and sex using the 2010 Brazilian population.

For each period and outcome, adjusted rates were classified using the Jenks natural breaks method. Health regions with invalid codes or no defined municipalities were excluded [39].

### 2.8 Ethical Considerations

SIM and SIH‐SUS are public, anonymised databases made available and freely accessible, provided by the Brazilian Ministry of Health.

The study complied with Law 14,874 (of 28 May 2024) on research involving human subjects in Brazil and with the Brazilian General Data Protection Law (Law 13.709/2018). Consequently, approval by an ethical review board was not required.

## 3 Results

### 3.1 Overall Mortality (SIM)

**FIGURE 2:**
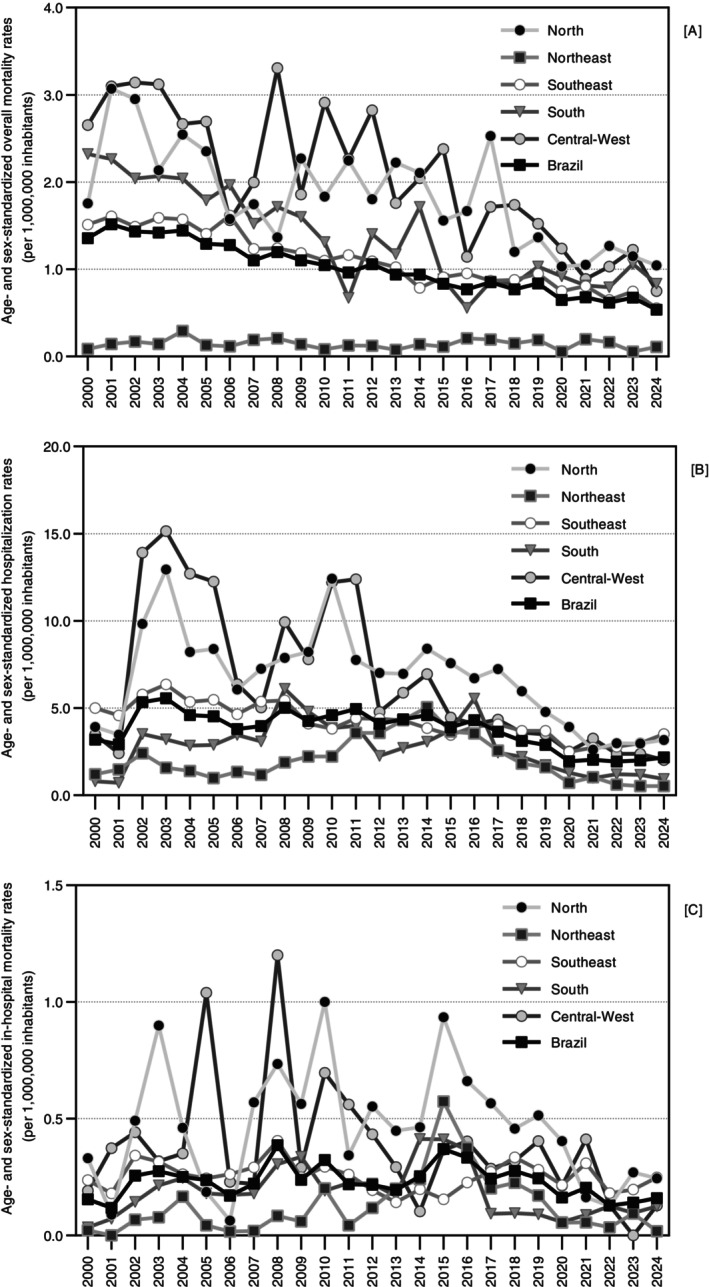
Age‐ and sex‐standardised rates related to paracoccidioidomycosis (per 1,000,000 inhabitants), by Brazilian macro‐region, 2000–2024. (A) Overall mortality (SIM), by region and national average. (B) Hospitalisation (SIH‐SUS), by region and national average. (C) In‐hospital mortality, by region and national average.

Between 2000 and 2024, 30,488,786 deaths were recorded in Brazil; 4904 (0.02%) mentioned PCM, including 2980 (60.8%) as the underlying cause of death. This corresponds to an age‐ and sex‐standardised mortality rate of 1.00 per 1,000,000 inhabitants (95% CI 0.97–1.02). The highest rates over the series were observed in the Central‐West and North (Figure 2A) and 29.9% of Brazilian municipalities recorded deaths involving PCM.

When PCM was the underlying cause of death, pulmonary PCM was the most frequently cited form, whereas when PCM was not the underlying cause, unspecified PCM predominated as an associated cause (Table S1).

When PCM was not the underlying cause, chronic obstructive pulmonary disease was the most frequent underlying cause, whereas septicaemia was the most frequent associated cause when PCM was the underlying cause (Table S1).

Most deaths had PCM as the underlying cause (60.8%), HIV/AIDS coinfection was recorded in 4.5% of cases and the vast majority occurred in hospitals and in men (Table 1).

**TABLE 1:** Numbers, proportions, crude rates and age‐ and sex‐standardised rates (per 1,000,000 inhabitants) of paracoccidioidomycosis‐related mortality, hospitalisation and in‐hospital mortality, by sociodemographic variables, Brazil, 2000–2024.

Indicator/variable	Mortality			Hospitalisation			In‐hospital mortality		
	*N* (%)	Crude rate (per 1,000,000 inhabitants)	Standardised rate (per 1,000,000 inhabitants) (95% CI)	*N* (%)	Crude rate (per 1,000,000 inhabitants)	Standardised rate (per 1,000,000 inhabitants) (95% CI)	*N* (%)	Crude rate (per 1,000,000 inhabitants)	Standardised rate (per 1,000,000 inhabitants) (95% CI)
Brazil—Total	4904 (100.0)	1.01	1 (0.97;1.02)	18,239 (100.0)	3.74	3.71 (3.66;3.77)	1136 (100.0)	0.23	0.23 (0.22;0.24)
Cause of hospital admission									
Primary	—	—	—	17,056 (93.5)	—	—	962 (84.7)	—	—
Secondary	—	—	—	1183 (6.5)	—	—	174 (15.3)	—	—
Death during hospitalisation									
No	—	—	—	17,103 (93.8)	—	—	0 (0.0)	—	—
Yes	—	—	—	1136 (6.2)	—	—	1136 (100.0)	—	—
Cause of death									
Underlying	2980 (60.8)	—	—	—	—	—	—	—	—
Associated	1924 (39.2)	—	—	—	—	—	—	—	—
HIV/AIDS coinfection									
Yes	220 (4.5)	—	—	138 (0.8)	—	—	8 (0.7)	—	—
No	4684 (95.5)	—	—	18,101 (99.2)	—	—	1128 (99.3)	—	—
Place where death occurred									
Hospital	4255 (86.8)	—	—	—	—	—	—	—	—
Other	180 (3.7)	—	—	—	—	—	—	—	—
Home	426 (8.7)	—	—	—	—	—	—	—	—
Public roads	36 (0.7)	—	—	—	—	—	—	—	—
Missing data	7 (0.1)	—	—	—	—	—	—	—	—
Sex									
Female	671 (13.7)	0.27	0.27 (0.25;0.29)	4381 (24.0)	1.76	1.75 (1.69;1.80)	279 (24.6)	0.11	0.11 (0.10;0.12)
Male	4232 (86.3)	1.77	1.76 (1.71;1.81)	13,858 (76.0)	5.80	5.77 (5.67;5.87)	857 (75.4)	0.36	0.36 (0.33;0.38)
Missing data	1 (0.0)	—	—	0 (0.0)	—	—	0 (0.0)	—	—
Age group									
≤ 14	58 (1.2)	0.05	0.05 (0.04;0.06)	2120 (11.6)	1.76	1.80 (1.72;1.87)	25 (2.2)	0.02	0.02 (0.01;0.03)
15–29	259 (5.3)	0.20	0.21 (0.18;0.23)	2156 (11.8)	1.66	1.71 (1.64;1.79)	88 (7.7)	0.07	0.07 (0.06;0.08)
30–39	348 (7.1)	0.46	0.45 (0.40;0.50)	1962 (10.8)	2.61	2.57 (2.46;2.68)	78 (6.9)	0.10	0.10 (0.08;0.12)
40–49	845 (17.2)	1.34	1.31 (1.22;1.40)	3598 (19.7)	5.72	5.61 (5.42;5.79)	178 (15.7)	0.28	0.28 (0.24;0.32)
50–59	1273 (26.0)	2.73	2.61 (2.47;2.75)	3860 (21.2)	8.27	7.92 (7.67;8.17)	244 (21.5)	0.52	0.50 (0.44;0.56)
60–69	1137 (23.2)	3.95	3.54 (3.34;3.75)	2509 (13.8)	8.72	7.82 (7.52;8.13)	249 (21.9)	0.87	0.78 (0.68;0.87)
≥ 70	982 (20.0)	4.19	3.79 (3.55;4.02)	2034 (11.2)	8.69	7.85 (7.50;8.19)	274 (24.1)	1.17	1.06 (0.93;1.18)
Missing data	2 (0.0)	—	—	0 (0.0)	—	—	0 (0.0)	—	—
Ethnicity^a^									
Caucasian	2876 (58.6)	1.27	—	4057 (22.2)	1.79	—	278 (24.5)	0.12	—
Afro–Brazilian/Afro–descendant	376 (7.7)	1.05	—	588 (3.2)	1.64	—	41 (3.6)	0.11	—
Asian–descendant	15 (0.3)	0.28	—	164 (0.9)	3.12	—	21 (1.8)	0.40	—
Mixed/Brazilian Pardo	1389 (28.3)	0.67	—	4496 (24.7)	2.17	—	312 (27.5)	0.15	—
Indigenous (Amerindians)	29 (0.6)	1.41	—	45 (0.2)	2.19	—	3 (0.3)	0.15	—
Missing data	219 (4.5)	—	—	8889 (48.7)	—	—	481 (42.3)	—	—
Region of residence									
North	582 (11.9)	1.43	1.77 (1.62;1.91)	2225 (12.2)	5.48	6.44 (6.17;6.71)	149 (13.1)	0.37	0.45 (0.38;0.53)
Northeast	184 (3.8)	0.14	0.14 (0.12;0.17)	2686 (14.7)	1.98	2.04 (1.96;2.11)	166 (14.6)	0.12	0.13 (0.11;0.15)
Southeast	2412 (49.2)	1.17	1.09 (1.05;1.14)	9048 (49.6)	4.41	4.22 (4.14;4.31)	555 (48.9)	0.27	0.25 (0.23;0.27)
South	1042 (21.2)	1.49	1.34 (1.26;1.42)	2061 (11.3)	2.95	2.73 (2.61;2.85)	142 (12.5)	0.20	0.19 (0.16;0.22)
Central–West	684 (13.9)	1.91	1.97 (1.83;2.12)	2219 (12.2)	6.18	6.24 (5.98;6.50)	124 (10.9)	0.35	0.36 (0.30;0.43)
Size of municipality									
Small I	1168 (23.8)	1.48	1.37 (1.29;1.45)	4115 (22.6)	5.22	5.05 (4.90;5.21)	233 (20.5)	0.30	0.28 (0.24;0.31)
Small II	1023 (20.9)	1.30	1.29 (1.22;1.37)	3846 (21.1)	4.87	4.88 (4.72;5.03)	199 (17.5)	0.25	0.25 (0.22;0.29)
Medium	710 (14.5)	1.23	1.25 (1.16;1.34)	2259 (12.4)	3.92	3.95 (3.78;4.11)	134 (11.8)	0.23	0.24 (0.20;0.28)
Large	1996 (40.7)	0.73	0.74 (0.71;0.77)	8019 (44.0)	2.95	2.94 (2.87;3.00)	570 (50.2)	0.21	0.21 (0.19;0.23)
Missing data	0	0.00	—	0 (0.0)	—		0 (0.0)	—	—
IBP									
Very low	1087 (22.2)	1.22	1.10 (1.03;1.16)	3556 (19.5)	3.98	3.71 (3.58;3.83)	240 (21.1)	0.27	0.24 (0.21;0.27)
Low	864 (17.6)	0.97	0.94 (0.87;1.00)	3075 (16.9)	3.44	3.35 (3.23;3.46)	180 (15.8)	0.20	0.20 (0.17;0.22)
Medium	1091 (22.2)	1.11	1.11 (1.04;1.17)	3734 (20.5)	3.81	3.77 (3.65;3.89)	261 (23.0)	0.27	0.27 (0.23;0.30)
High	1296 (26.4)	1.33	1.35 (1.28;1.43)	4409 (24.2)	4.53	4.57 (4.43;4.70)	297 (26.1)	0.31	0.31 (0.28;0.35)
Very high	559 (11.4)	0.49	0.53 (0.49;0.58)	3464 (19.0)	3.06	3.19 (3.08;3.29)	158 (13.9)	0.14	0.15 (0.13;0.17)
Missing data	7 (0.1)	—	—	1 (0.0)	—		0 (0.0)	—	—
Typology of municipality									
Urban	3532 (72.0)	0.96	0.96 (0.92;0.99)	13,170 (72.2)	3.56	3.53 (3.47;3.59)	875 (77.0)	0.24	0.24 (0.22;0.25)
Intermediate adjacent	399 (8.1)	1.25	1.22 (1.10;1.34)	1336 (7.3)	4.20	4.17 (3.94;4.39)	70 (6.2)	0.22	0.21 (0.16;0.27)
Intermediate remote	73 (1.5)	2.18	2.70 (2.07;3.34)	216 (1.2)	6.46	7.34 (6.34;8.34)	13 (1.1)	0.39	0.49 (0.21;0.76)
Rural adjacent	737 (15.0)	1.01	0.96 (0.89;1.03)	2991 (16.4)	4.08	4.02 (3.87;4.16)	146 (12.9)	0.20	0.19 (0.16;0.22)
Rural remote	156 (3.2)	1.73	2.00 (1.68;2.32)	525 (2.9)	5.83	6.57 (6.00;7.14)	32 (2.8)	0.36	0.41 (0.27;0.55)
Missing data	7 (0.1)	—	—	1 (0.0)	—		0 (0.0)	—	—

Abbreviations: %, percentage; —, not calculated; IBP, Brazilian Index of Deprivation (*Índice Brasileiro de Privação*); *N*, number.

^a^ Self‐reported ethnicity data available from 2008 for hospitalisations.

Highest rates were observed in those aged ≥ 70 years, indigenous people, Central‐West, municipalities with up to 20,000 inhabitants, municipalities with high deprivation and intermediate‐remote municipalities (Table 1).

Time‐trend analysis showed a significant reduction in PCM mortality in Brazil (AAPC = −3.85; 95% CI, −4.31 to −3.45). The decline was steeper among men, adults aged 40–59 years, Caucasians, and residents in the South, whereas the Northeast showed no statistically significant trend over the whole period (Table 2).

**TABLE 2:** Temporal trends in paracoccidioidomycosis‐related mortality, hospitalisation and in‐hospital mortality (per 1,000,000 inhabitants), according to Joinpoint regression, by sociodemographic variables, Brazil, 2000–2024.

Indicator/variables	Mortality			Hospitalisation			In‐hospital mortality		
	Period	APC (95% CI)	AAPC (95% CI)	Period	APC (95% CI)	AAPC (95% CI)	Period	APC (95% CI)	AAPC (95% CI)
Brazil—Total	2000–2024	−3.85* (−4.31;−3.45)	−3.85* (−4.31;−3.45)	2000–2002	30.66 (−0.13;69.81)	−2.83* (−4.60;−1.22)	2000–2016	2.57* (0.14;13.60)	−0.66 (−2.55;1.26)
				2002–2015	−1.03 (−19.20;1.21)		2016–2024	−9.91* (−30.53;−2.77)	
				2015–2024	−9.39 (−21.25;1.64)				
Sex									
Female	2000–2024	−2.51* (−4.10;−1.01)	−2.51* (−4.10;−1.01)	2000–2013	5.82* (2.31;11.76)	−2.15 (−5.48;0.93)	2000–2015	6.41* (2.30;27.94)	0.25 (−2.73;3.60)
				2013–2024	−12.92* (−19.78;−8.97)		2015–2024	−10.99* (−33.97;−3.05)	
Male	2000–2024	−4.05* (−4.46;−3.70)	−4.05* (−4.46;−3.70)	2000–2002	28.33 (−1.37;67.27)	−3.03* (−4.29;−1.92)	2000–2018	0.94 (−1.16;60.77)	−1.04 (−2.84;0.78)
				2002–2016	−2.22 (−21.49;0.58)		2018–2024	−12.29* (−44.23;−1.58)	
				2016–2024	−8.37 (−28.11;7.06)				
Age group									
≤ 14	2000–2024	−1.97 (−6.51;1.56)	−1.97 (−6.51;1.56)	2000–2011	15.98* (10.67;23.49)	−1.07 (−5.26;2.90)	2000–2024	−2.57 (−6.99;1.04)	−2.57 (−6.99;1.04)
				2011–2021	−16.21* (−29.30;−11.49)				
				2021–2024	27.31 (−8.22;105.59)				
15–29	2000–2024	−1.35 (−3.32;0.47)	−1.35 (−3.32;0.47)	2000–2024	−0.74 (−2.09;0.56)	−0.74 (−2.09;0.56)	2000–2024	−0.41 (−2.81;2.01)	−0.41 (−2.81;2.01)
30–39	2000–2024	−6.08* (−8.28;−4.42)	−6.08* (−8.28;−4.42)	2000–2002	30.57* (0.29;67.39)	−5.57* (−6.88;−4.49)	2000–2006	13.07 (−6.19;114.77)	−7.02* (−11.24;−4.22)
				2002–2024	−6.38* (−8.17;−5.55)		2006–2024	−11.03* (−48.06;−7.86)	
40–49	2000–2003	5.94 (−6.59;28.90)	−6.40* (−7.71;−5.38)	2000–2002	25.74 (−5.59;69.26)	−6.23* (−7.55;−5.16)	2000–2024	−3.88* (−6.96;−1.21)	−3.88* (−6.96;−1.21)
	2003–2024	−7.12* (−15.56;−5.99)		2002–2024	−7.03* (−23.05;−6.08)				
50–59	2000–2013	−3.85 (−5.32;1.60)	−5.69* (−6.97;−4.52)	2000–2003	19.73* (6.83;53.15)	−4.71* (−6.17;−3.42)	2000–2010	3.25 (−3.15;85.94)	−3.51* (−6.08;−1.03)
	2013–2024	−8.67* (−17.53;−6.55)		2003–2016	−3.68* (−5.39;−2.00)		2010–2024	−7.51* (−39.30;−3.63)	
				2016–2024	−12.18* (−19.07;−9.34)				
60–69	2000–2024	−5.49* (−6.48;−4.60)	−5.49* (−6.48;−4.60)	2000–2002	26.69 (−5.56;74.71)	−3.46* (−5.01;−1.91)	2000–2024	−1.20 (−4.14;2.31)	−1.20 (−4.14;2.31)
				2002–2016	−2.30 (−23.76;11.18)				
				2016–2024	−8.65 (−30.36;17.25)				
≥ 70	2000–2024	−5.62* (−6.79;−4.60)	−5.62* (−6.79;−4.60)	2000–2014	5.57* (2.48;11.02)	−2.91 (−6.82;1.25)	2000–2015	9.18* (5.65;16.19)	−0.18 (−3.79;4.38)
				2014–2024	−16.76* (−23.78;−12.82)		2015–2024	−16.37* (−26.05;−11.20)	
Ethnicity^a^									
Caucasian	2000–2024	−4.08* (−4.82;−3.47)	−4.08* (−4.82;−3.47)	—	—	—	—	—	—
Afro–Brazilian/Afro–descendant	2000–2024	−3.83* (−5.60;−2.17)	−3.83* (−5.60;−2.17)	—	—	—	—	—	—
Asian–descendant	2000–2024	−2.58 (−6.55;0.93)	−2.58 (−6.55;0.93)	—	—	—	—	—	—
Mixed/Pardo Brazilians	2000–2024	−1.85* (−2.65;−1.06)	−1.85* (−2.65;−1.06)	—	—	—	—	—	—
Indigenous (Amerindians)	2000–2024	−2.62 (−6.62;1.52)	−2.62 (−6.62;1.52)	—	—	—	—	—	—
Region of residence									
North	2000–2024	−3.25* (−4.78;−1.86)	−3.25* (−4.78;−1.86)	2000–2002	73.53* (6.02;142.78)	−3.47* (−5.66;−1.53)	2000–2015	2.14 (−2.08;103.09)	−2.20 (−5.34;0.93)
				2002–2016	−2.03 (−5.58;3.85)		2015–2024	−12.12* (−53.84;−2.92)	
				2016–2024	−11.81* (−33.23;−5.79)				
Northeast	2000–2024	−0.71 (−3.55;2.18)	−0.71 (−3.55;2.18)	2000–2006	−4.68 (−20.04;4.55)	1.00 (−3.77;6.45)	2000–2015	18.31* (12.54;35.30)	4.58 (−1.27;13.59)
				2006–2014	19.61* (14.72;31.79)		2015–2024	−22.61* (−42.82;−15.04)	
				2014–2024	−22.14* (−26.12;−19.24)				
Southeast	2000–2024	−3.88* (−4.50;−3.33)	−3.88* (−4.50;−3.33)	2000–2003	6.07 (−2.98;23.97)	−2.90* (−3.72;−2.16)	2000–2024	−0.80 (−2.50;0.92)	−0.80 (−2.50;0.92)
				2003–2024	−3.38* (−10.48;−2.58)				
South	2000–2024	−4.62* (−5.85;−3.63)	−4.62* (−5.85;−3.63)	2000–2002	140.04* (2.96;356.39)	−2.72 (−6.66;0.63)	2000–2016	6.64 (−2.24;19.18)	−0.98 (−5.29;3.28)
				2002–2016	1.04 (−9.30;6.28)		2016–2019	−44.65 (−59.85;17.65)	
				2016–2024	−19.17* (−37.77;−10.77)		2019–2024	14.05 (−18.15;111.91)	
Central–West	2000–2012	−1.50 (−4.26;23.31)	−4.28* (−5.89;−2.92)	2000–2002	143.60* (26.66;289.45)	−6.76* (−9.78;−4.58)	2000–2005	32.66 (−1.45;257.63)	−4.05* (−8.53;−0.22)
	2012–2024	−7.73* (−35.76;−4.68)		2002–2024	−7.88* (−10.49;−6.30)		2005–2024	−7.52* (−32.03;−4.26)	
Size of municipality									
Small I	2000–2024	−2.98* (−4.38;−1.78)	−2.98* (−4.38;−1.78)	2000–2011	5.08* (0.42;20.33)	−1.90 (−4.07;0.08)	2000–2008	12.59* (5.58;31.46)	−0.90 (−3.04;1.22)
				2011–2024	−7.66* (−16.02;−4.17)		2008–2024	−4.64* (−8.06;−2.50)	
Small II	2000–2024	−2.99* (−3.93;−2.17)	−2.99* (−3.93;−2.17)	2000–2014	2.56* (0.59;5.48)	−2.42* (−4.32;−0.67)	2000–2015	8.08* (4.12;22.62)	1.57 (−1.40;5.14)
				2014–2024	−12.26* (−17.87;−9.12)		2015–2024	−10.42* (−27.27;−2.56)	
Medium	2000–2003	8.66 (−6.44;52.71)	−5.58* (−7.23;−4.34)	2000–2002	68.27* (16.14;132.89)	−4.26* (−6.01;−2.78)	2000–2003	45.72 (−4.78;314.15)	−2.05 (−4.93;0.73)
	2003–2024	−6.46* (−27.42;−4.27)		2002–2024	−5.26* (−6.88;−4.20)		2003–2024	−3.21 (−40.73;3.42)	
Large	2000–2024	−3.91* (−4.73;−3.21)	−3.91* (−4.73;−3.21)	2000–2010	1.16 (−2.28;21.66)	−2.92* (−4.37;−1.59)	2000–2016	2.21 (−0.39;13.60)	−0.90 (−3.46;1.71)
				2010–2024	−5.63* (−16.77;−3.43)		2016–2024	−10.36* (−35.41;−3.21)	
IBP									
Very low	2000–2024	−4.54* (−5.50;−3.71)	−4.54* (−5.50;−3.71)	2000–2002	30.46 (−2.01;63.24)	−2.29* (−3.40;−1.23)	2000–2024	−1.04 (−3.83;1.80)	−1.04 (−3.83;1.80)
				2002–2024	−2.93* (−7.23;−1.85)				
Low	2000–2024	−5.08* (−5.99;−4.36)	−5.08* (−5.99;−4.36)	2000–2011	−0.16 (−2.33;5.17)	−3.59* (−4.87;−2.50)	2000–2024	−1.35 (−4.11;1.36)	−1.35 (−4.11;1.36)
				2011–2024	−6.71* (−10.77;−4.83)				
Medium	2000–2024	−3.39* (−4.35;−2.57)	−3.39* (−4.35;−2.57)	2000–2003	20.88 (−0.66;52.82)	‐2.57* (−3.80;−1.41)	2000–2024	−1.12 (−3.76;1.47)	−1.12 (−3.76;1.47)
				2003–2024	−3.57* (−5.99;−2.50)				
High	2000–2024	−3.95* (−4.78;−3.24)	−3.95* (−4.78;−3.24)	2000–2002	71.54* (6.32;173.72)	−4.15* (−6.53;−2.18)	2000–2015	5.65* (1.72;17.93)	−0.37 (−3.55;3.02)
				2002–2024	−5.16* (−7.95;−3.85)		2015–2024	−13.01* (−32.51;−5.52)	
Very high	2000–2024	−1.35 (−2.76;0.02)	−1.35 (−2.76;0.02)	2000–2014	7.04* (5.03;9.83)	−1.11 (−4.99;2.72)	2000–2015	8.44* (3.23;33.17)	1.01 (−3.49;6.46)
				2014–2024	−17.04* (−21.62;−14.01)		2015–2024	−15.80* (−51.70;−4.53)	
Typology of municipality									
Urban	2000–2024	−4.24* (−4.72;−3.82)	−4.24* (−4.72;−3.82)	2000–2002	29.87* (0.39;62.75)	−3.15* (−4.48;−1.96)	2000–2016	2.45 (−0.02;11.31)	−0.76 (−3.18;1.68)
				2002–2016	−2.14* (−5.46;−0.10)		2016–2024	−10.22* (−30.46;−3.28)	
				2016–2024	−9.19* (−23.00;−5.26)				
Intermediate adjacent	2000–2024	−2.97* (−4.56;−1.57)	−2.97* (−4.56;−1.57)	2000–2003	22.17 (−1.93;88.26)	−3.11* (−4.90;−1.54)	2000–2024	−0.43 (−4.06;3.44)	−0.43 (−4.06;3.44)
				2003–2024	−4.25* (−11.06;−3.00)				
Intermediate remote	2000–2024	−1.98 (−5.15;1.31)	−1.98 (−5.15;1.31)	2000–2024	−1.38 (−4.68;2.01)	−1.38 (−4.68;2.01)	2000–2024	0.17 (−3.29;4.03)	0.17 (−3.29;4.03)
Rural adjacent	2000–2024	−3.06* (−4.67;−1.69)	−3.06* (−4.67;−1.69)	2000–2014	6.98* (4.02;12.00)	−0.99 (−4.50;2.41)	2000–2010	12.60* (4.22;66.20)	0.02 (−2.64;2.75)
				2014–2024	−15.28* (−23.13;−10.92)		2010–2024	−5.42* (−14.62;−1.41)	
Rural remote	2000–2024	−0.62 (−3.26;2.16)	−0.62 (−3.26;2.16)	2000–2002	64.51 (−5.92;219.49)	−4.04* (−6.64;−1.87)	2000–2024	−2.13 (−6.43;1.83)	−2.13 (−6.43;1.83)
				2002–2024	−4.93* (−32.36;−1.03)				

Abbreviations: —, not calculated; 95% CI, 95% confidence intervals; AAPC, average annual percent change; APC, annual percent change; IBP, Brazilian Index of Deprivation (*Índice Brasileiro de Privação*).

^a^ Self‐reported ethnicity data available from 2008 for hospitalisations.

* Significantly different from 0 (*p* < 0.05).

**FIGURE 3:**
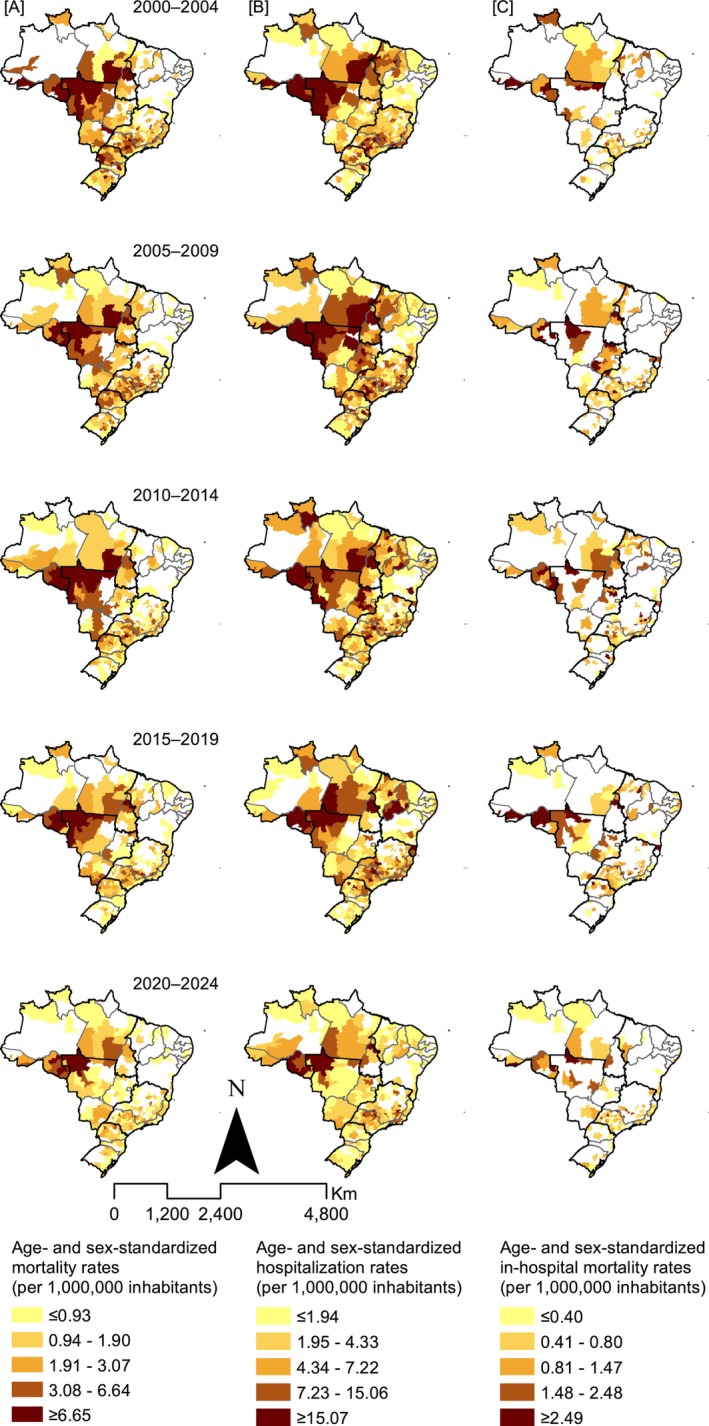
Distribution of age‐ and sex‐standardised rates related to paracoccidioidomycosis (per 1,000,000 inhabitants), by health region, Brazil, 2000–2024: (A) overall mortality (SIM); (B) hospitalisation (SIH‐SUS); and (C) in‐hospital mortality.

The spatial distribution of adjusted mortality rates was heterogeneous, with clusters of high rates in health regions of Rondônia and northern Mato Grosso and, up to 2019, in areas of southern Pará. Rates above 3.08 per 1,000,000 were identified in São Paulo, Parana, Espirito Santo, southern Pará, southern Minas Gerais and northern Tocantins States, revealing extense endemic areas (Figure 3A).

### 3.2 Hospital Admissions (SIH‐SUS)

From 2000 to 2024, a total of 543,535 AIH were recorded in SIH‐SUS; of these, 18,239 (0.006%) mentioned PCM, corresponding to a standardised hospitalisation rate of 3.71 per 1,000,000 inhabitants. The highest rates over the series were observed in the Central‐West and North regions (Figure 2B). 48.5% of Brazilian municipalities reported hospital admissions for the specified PCM.

When PCM was the primary diagnosis for admission, unspecified PCM was the most frequently cited form, a pattern similar to that observed as a secondary diagnosis when PCM was not the primary diagnosis (Table S1).

When PCM was not the primary diagnosis, chronic obstructive pulmonary disease was the most frequent primary diagnosis. Among admissions with PCM as the primary diagnosis, chronic Chagas disease with cardiac involvement was the most frequent secondary diagnosis (Table S1).

Most admissions had PCM recorded as the primary diagnosis (93.5%), indicating that PCM often constituted the central reason for hospitalisation. In 6.2% of admissions, the outcome was death and 0.8% had HIV/AIDS coinfection (Table 1).

The highest rates were observed in men, adults aged 50 years or over, residents of the North region, municipalities with up to 20,000 inhabitants, municipalities with high deprivation and intermediate‐remote municipalities (Table 1).

PCM hospitalisation rates showed a significant downward trend nationwide (AAPC = −2.83; 95% CI, −4.60 to −1.22). The most pronounced reduction occurred among adults aged 40–49 years, whereas trends were less clearly defined for children, young adults and older adults over the whole period (Table 2).

The Central‐West showed an initial increase in admissions followed by a marked decline. A similar pattern was observed in the Southeast, whereas the North showed an initial increase and a decline from the mid‐2010s onwards. The most significant reduction occurred in medium‐sized municipalities and in municipalities with high deprivation (Table 2).

The spatial distribution of PCM hospitalisations was markedly heterogeneous, with the highest adjusted rates concentrated in the health regions of Rondônia and northern Mato Grosso, and between 2000 and 2019, in areas of southern Pará and northern Tocantins (Figure 3B).

### 3.3 In‐Hospital Mortality (SIH‐SUS)

Of the total of AIH recorded in the period, 3.9% (11,367,369) resulted in death. Among hospital admissions ending in death, 1136 (0.01%) mentioned PCM, corresponding to a standardised in‐hospital mortality rate of 0.23 per 1,000,000 inhabitants. The highest rates were observed in the Central‐West and North (Figure 2C) and 11.3% of Brazilian municipalities recorded such admissions.

When PCM was the primary diagnosis among in‐hospital deaths, unspecified pulmonary blastomycosis was the most frequent PCM‐related code. As a secondary diagnosis, when PCM was not the primary diagnosis, disseminated PCM was the most frequent code (Table S1).

When PCM was not the primary diagnosis among in‐hospital deaths, septicaemia was the most frequent primary diagnosis, while chronic Chagas disease with cardiac involvement predominated as a secondary diagnosis when PCM was the primary diagnosis (Table S1).

Most admissions that evolved to death had PCM as the primary diagnosis (84.7%). HIV/AIDS coinfection was recorded in 0.7% of these AIH, and the highest rates were observed in men, adults aged 50 years or over, residents of the North region, municipalities with up to 20,000 inhabitants, municipalities with high deprivation and intermediate‐remote municipalities (Table 1).

Time‐trend analysis of in‐hospital mortality revealed an initial increase followed by a decline. Rates increased significantly between 2000 and 2016 and decreased between 2016 and 2024, but the overall AAPC was not statistically significant, indicating no clear long‐term trend (Table 2).

The most significant reduction over the period was observed among individuals aged 30–39 years. Reductions were also observed in the 40–49 and 50–59 year age groups, and only the Central‐West showed a significant decline over the whole period. No consistent significant trends were identified by municipality size, IBP or typology (Table 2).

The spatial distribution of in‐hospital mortality was also heterogeneous, with the highest adjusted rates concentrated in health regions of Rondônia and northern Mato Grosso (Figure 3C).

## 4 Discussion

This nationwide study provides an updated overview of the burden and inequalities of PCM in Brazil. By integrating mortality, hospital admissions and in‐hospital mortality over 25 years, it refines the understanding of PCM as a neglected systemic mycosis and a persistent public health problem [2, 15, 16, 26, 27, 40]. In the Northeast region, the low PCM rate observed across the series may be partly explained by the semi‐arid climate in many municipalities. Our findings extend previous historical series and are consistent with recent reviews on the epidemiology and clinical management of the disease [1, 2, 3, 4, 13, 40], reinforcing the need to integrate PCM into surveillance, care, and NTD policy agendas in Brazil [8, 9, 15, 16].

Brazil has been consolidating a specific agenda for NTDs, with the production of national epidemiological bulletins; however, these bulletins do not include systemic mycoses such as PCM [15, 16].

Although PCM is not formally included in the Brazilian national list of compulsorily notifiable diseases [11], it shares the epidemiological and social profile of NTDs prioritised by the Ministry of Health [15, 16]. It disproportionately affects populations living in rural and agricultural settings, who are exposed to environmental transformation, delayed diagnosis and barriers to specialised care [13, 14, 17, 18, 19, 20].

The declining trends in mortality and hospitalisation suggest some progress in access to diagnosis and treatment in certain territories and population groups. At the same time, the absence of a clear overall decline in in‐hospital mortality indicates that severe cases continue to arrive late or under complex clinical conditions, despite the availability of effective therapeutic regimens [2]. The frequent co‐recording of chronic obstructive pulmonary disease, chronic Chagas cardiomyopathy and septicaemia supports the role of multimorbidity and advanced disease among severe presentations [41]. This contrast reflects differences in the natural histories and care pathways of the two conditions and also highlights structural limitations of SIH‐SUS, which was primarily designed to finance and manage admissions and thus has low sensitivity in capturing the full burden of chronic, long‐duration diseases [27, 42].

Our results are consistent with classical PCM mortality studies that already showed a greater burden among adult men and in endemic areas of the Southeast, South and Central‐West [18, 22, 43]. They also align with analyses of hospital morbidity, which identified PCM as one of the main systemic mycoses in terms of hospitalisations [21, 40].

More recent studies have documented emerging hotspots linked to road works, environmental disturbance, deforestation, agribusiness expansion and climatic variability in Rio de Janeiro, the Amazon region, Mato Grosso do Sul and the Cerrado [14, 17, 19]. By extending the series through 2024 and including the COVID‐19 period, our study shows continuity rather than disappearance of the problem [44].

Taken together, our findings show that PCM remains concentrated in specific territories characterised by persistent high‐risk areas and emerging clusters in agricultural frontier regions. This is evidenced by spatio‐temporal analyses of acute/subacute forms in the Amazon and Central‐West regions [14, 19], areas that have municipalities with a high proportion of indigenous population [45], reinforcing the findings of higher mortality rates in this study. These patterns reinforce that PCM is embedded in the ‘social reality’ of certain regions and cannot be considered a residual or disappearing problem [2, 21, 22].

High rates in Rondônia and northern Mato Grosso, as well as clusters in other parts of the Central‐West and Legal Amazon, align with evidence linking PCM to deforestation, agricultural frontier expansion and other land‐use changes [11, 12, 13, 14, 19, 31, 46]. These territories combine environmental disturbance, occupational soil exposure and social vulnerability, which likely sustain both persistent and emerging hyperendemic areas.

The higher burden among men, particularly those aged 50–59 years, is consistent with the predominance of chronic forms among rural workers with prolonged exposure to soil to agricultural and soil‐handling activities [2, 3, 6, 7, 13, 19, 21, 22, 43]. This concentration of mortality and hospitalisations in this age group highlights the impact of PCM on the economically active population and underscores the potential for disability, loss of income and long‐term social impact. In addition, the distribution of *P. lutzii* in the Central‐West and Amazon may complicate serological diagnosis because of lower cross‐reactivity with antigens from other Paracoccidioides species [1, 2].

HIV/AIDS coinfection was infrequent in our datasets, but this finding should be interpreted cautiously. Prophylactic use of sulfamethoxazole‐trimethoprim in advanced HIV may reduce overt PCM, while published series indicate that when coinfection occurs, it is usually severe and may present as disseminated or mixed disease [2, 47, 48, 49].

In endemic areas, HIV infection should therefore be actively investigated when PCM is diagnosed, and management should be closely coordinated with specialised infectious diseases services [2].

The strong association between PCM and social inequalities emerged consistently in our analyses. Higher rates in small municipalities, areas with high deprivation and territories with rural or intermediate characteristics mirror the pattern seen for other endemic NTDs in Brazil, such as Chagas disease, leprosy, schistosomiasis, leishmaniases and snakebites [15, 16, 41, 50]. These results show that the burden of PCM is concentrated in contexts characterised by poverty, low schooling, inadequate sanitation and high dependence on agricultural labour, reinforcing its status as a socially determined disease [2, 15, 36, 37]. At the same time, the proportionally steeper reductions in small municipalities (up to 50,000 inhabitants) and in more deprived areas may reflect localised improvements in living and working conditions [36, 37, 46]. Still, they may also indicate a shift in risk to new agricultural frontier areas, with migrant workers moving to recently deforested regions [14, 19, 31].

Regarding ethnic inequalities, although SIM allows detection of higher mortality from several NTDs among Afro‐Brazilian and mixed‐ethnicity populations [15, 16]. The high proportion of missing ethnicity data in SIH‐SUS limited deeper analysis of ethnic inequities. Improving completeness of ethnicity recording is important for identifying marginalised groups and for planning specific surveillance and care strategies [25, 27, 37].

From a surveillance perspective, our results strengthen the argument for stable national notification. PCM fulfils classical public health criteria for notification and Brazilian guidelines have long recommended compulsory reporting and a national case registry [2, 8, 9, 11, 12, 15, 16]. State‐level experiences have demonstrated their technical feasibility and usefulness [11, 12, 21, 24].

In this light, the inclusion of PCM in Brazil's national list of notifiable diseases would represent a step forward, consistent with accumulated clinical and epidemiological knowledge and with international recognition of PCM as a priority endemic mycosis [2, 8, 9, 12, 24]. The subsequent revocation of this measure at the national level, without a widely discussed technical‐scientific justification, has created a misalignment between the magnitude of disease burden, the consensus reflected in guidelines and epidemiological analyses, and surveillance policy [12, 24]. The absence of PCM from the current national list of notifiable diseases should be contextualised: PCM was included in the national list in February 2020 (Portaria GM/MS No 264/2020) but the measure was revoked in May 2020 (Portaria GM/MS No 1.061/2020) and PCM has not been included in subsequent updates (e.g., Portaria GM/MS No 6.734/2025) [51, 52]. Our findings point to the need for an integrated surveillance model that links SIM, SIH‐SUS, case notification systems and mechanisms to regulate access to medicines and hospital care, enabling monitoring of the entire care continuum from diagnosis to outcome [2, 12, 15, 16, 26, 27].

Public health implications extend beyond surveillance. The overlap between PCM burden, agribusiness expansion and deforestation supports its inclusion in the One Health agenda and in territorial planning and biome protection policies [8, 9, 12, 15, 16, 31]. Expanded diagnostic capacity, timely access to antifungal treatment and stronger referral networks in endemic and frontier regions remain essential [1, 2, 3, 53].

This study has limitations inherent to the use of nationwide secondary data. SIM and SIH‐SUS could not be linked at the individual level and SIH‐SUS does not distinguish repeated admissions for the same person [21, 22, 25, 26, 27, 42]. Completeness of variables such as ethnicity and comorbidities also varies across systems and over time.

Diagnostic misclassification, including historical inconsistencies in coding systemic mycoses, may have led to under‐ or overestimation of events attributed to PCM [33, 43, 54, 55]. The use of health regions reduced rate instability and aligned the analysis with SUS planning, but may have masked heterogeneity within regions.

Despite these limitations, our study has a nationwide coverage and a prolonged observation period. We further integrated analyses of three complementary outcomes (mortality, hospitalisation and in‐hospital mortality), used age‐ and sex‐standardised rates, and included social deprivation and municipal typology indicators. A spatio‐temporal approach based on health regions further enhanced the validity of the generated evidence [15, 16, 19, 21, 22, 30, 36, 37, 50]. The inclusion of the COVID‐19 period allows, albeit indirectly, assessment of potential pandemic‐related impacts on the care network and PCM‐related events [44]. Finally, by identifying priority areas and persistent inequality patterns, the study provides concrete inputs for planning surveillance, prevention, diagnosis and care actions, strengthening the technical case for the institution and consolidation of PCM as a notifiable disease in Brazil and for its prioritisation on the national NTD agenda within the SUS [2, 8, 9, 12, 15, 16].

## 5 Conclusions

The integrated analysis of PCM‐related mortality, hospitalisations and in‐hospital mortality in a 25‐year national time series confirms that it remains a relevant public health problem in Brazil. Our data reinforce that PCM is far from being ‘residual’ and will continue to generate severe cases, deaths and sequelae as long as social vulnerability, occupational exposure and environmental degradation persist. In addition, the emergence of *P. lutzii* has altered the epidemiological landscape and made serological diagnosis more challenging.

In light of these findings, PCM should be included in the national list of notifiable diseases and recognised and prioritised within Brazil's NTD and One Health agendas, with actions that integrate surveillance, clinical care and environmental policies. Stable instatement of compulsory national‐level notification, integrated with SIM, SIH‐SUS and regulation of access to diagnostics and medicines, is a strategic step to make the actual burden of disease visible, guide the organisation of care pathways and direct resources to the most affected areas and populations.

## Funding

The authors have nothing to report.

## Conflicts of Interest

The authors declare no conflicts of interest.

## Supporting information


**Table S1:** Mentions of paracoccidioidomycosis (B41) or the 10 leading ICD‐10 codes as underlying/primary or associated/secondary causes of mortality (SIM), hospital admissions and in‐hospital mortality (SIH‐SUS), Brazil, 2000–2024.

## Data Availability

All data used in this study are publicly available from the Department of Informatics of the Unified Health System (DATASUS) of the Brazilian Ministry of Health. Mortality data from SIM can be accessed via the FTP repository: ftp://ftp.datasus.gov.br/dissemin/publicos/SIM/CID10/DORES/. Hospital admission data from SIH‐SUS are available at: ftp://ftp.datasus.gov.br/dissemin/publicos/SIHSUS/. Both databases are anonymised, publicly available and freely downloadable without prior authorisation.

## References

[1] R. C. Hahn , F. Hagen , R. P. Mendes , et al., “Paracoccidioidomycosis: Current Status and Future Trends,” Clinical Microbiology Reviews 35, no. 4 (2022): e0023321, 10.1128/cmr.00233-21. 36074014 PMC9769695

[2] M. A. Shikanai‐Yasuda , R. P. Mendes , A. L. Colombo , et al., “Brazilian Guidelines for the Clinical Management of Paracoccidioidomycosis,” Revista da Sociedade Brasileira de Medicina Tropical 50, no. 5 (2017): 715–740, 10.1590/0037-8682-0230-2017. 28746570

[3] M. M. Costa and S. H. M. Silva , “Epidemiology, Clinical, and Therapeutic Aspects of Paracoccidioidomycosis,” Current Tropical Medicine Reports 1 (2014): 138–144, 10.1007/s40475-014-0013-z.

[4] G. R. Thompson , T. Le , A. Chindamporn , et al., “Global Guideline for the Diagnosis and Management of the Endemic Mycoses: An Initiative of the European Confederation of Medical Mycology in Cooperation With the International Society for Human and Animal Mycology,” Lancet Infectious Diseases 21, no. 12 (2021): e364–e374, 10.1016/S1473-3099(21)00191-2. 34364529 PMC9450022

[5] G. Benard , “Pathogenesis and Classification of Paracocidioidomycosis: New Insights From Old Good Stuff,” Open Forum Infectious Diseases 8, no. 3 (2020): ofaa624, 10.1093/ofid/ofaa624. 33728354 PMC7944344

[6] B. Wanke and M. A. Aidê , “Chapter 6—Paracoccidioidomycosis,” Jornal Brasileiro de Pneumologia 35, no. 12 (2009): 1245–1249, https://www.jornaldepneumologia.com.br/Content/imagebank/pdf/2009_35_12_13_english.pdf. 20126928 10.1590/s1806-37132009001200013

[7] K. L. Curtis , J. A. W. Gold , J. M. Ritter , et al., “Dermatologic Fungal Neglected Tropical Diseases—Part I. Epidemiology and Clinical Features,” Journal of the American Academy of Dermatology 92, no. 6 (2025): 1189–1206, 10.1016/j.jaad.2024.03.056. 38852743 PMC11970523

[8] WHO , “Ending the Neglect to Attain the Sustainable Development Goals: A Road Map for Neglected Tropical Diseases 2021–2030,” 2021, https://www.who.int/publications/i/item/9789240010352.

[9] WHO , Control of Neglected Tropical Diseases (World Health Organization, 2025), https://www.who.int/teams/control‐of‐neglected‐tropical‐diseases.

[10] WHO , WHO Fungal Priority Pathogens List to Guide Research, Development and Public Health Action (World Health Organization, 2022), https://iris.who.int/server/api/core/bitstreams/69af4379‐4f27‐4ac4‐8973‐74d7b52af7bd/content.

[11] Brasil , “Portaria GM/MS n° 6.734, de 18 de março de 2025. Portaria GM/MS N^o^ 6.734, 18 março 2025,” 2025, https://bvsms.saude.gov.br/bvs/saudelegis/gm/2025/prt6734_31_03_2025.html.

[12] M. A. Millington , S. A. Nishioka , S. T. Martins , Z. M. G. Santos , F. E. L. Ferreira Júnior , and R. V. Alves , “Paracoccidioidomycosis: Historical Approach and Perspectives for Implementation of Surveillance and Control,” Epidemiologia e Serviços de Saúde 27 (2018): e0500002, 10.5123/S1679-49742018000500002. 30133689

[13] R. Martinez , “Epidemiology of Paracoccidioidomycosis,” Revista do Instituto de Medicina Tropical de São Paulo 57, no. Suppl 19 (2015): 11–20, 10.1590/S0036-46652015000700004. 26465364 PMC4711199

[14] L. R. Fabris , N. G. de Oliveira , B. E. Bortolomai , et al., “The Effect of Geoclimatic Factors on the Distribution of Paracoccidioidomycosis in Mato Grosso do Sul, Brazil,” Journal of Fungi (Basel) 10, no. 3 (2024): 165, 10.3390/jof10030165. PMC10971066 38535174

[15] Brasil , Neglected Tropical Diseases in Brazil Morbidity, Mortality and National Response in the Context of the Sustainable Development Goals 2016–2020. Ministério da Saúde (Ministério da Saúde, Secretaria de Vigilância em Saúde e Ambiente, 2024), https://www.gov.br/saude/pt‐br/centrais‐de‐conteudo/publicacoes/boletins/epidemiologicos/especiais/2024/boletim‐epidemiologico‐de‐doencas‐tropicais‐negligenciadas‐numero‐especial‐jan‐2024.

[16] Brasil , Neglected Tropical Diseases, Impact on Child Morbidity and Mortality in Brazil 2010 to 2023. Ministério da Saúde (Secretaria de Vigilância em Saúde e Ambiente, Ministério da Saúde, 2025), https://www.gov.br/saude/pt‐br/centrais‐de‐conteudo/publicacoes/boletins/epidemiologicos/especiais/2025/boletim‐epidemiologico‐de‐doencas‐tropicais‐negligenciadas‐numero‐especial‐jan‐2025.pdf/view.

[17] E. M. M. Falcão , P. M. de Macedo , Z. F. Coutinho , F. I. Bastos , and A. C. F. do Valle , “Rising Rates of Paracoccidioidomycosis‐Related Hospitalizations and In‐Hospital Deaths, Rio de Janeiro, Brazil (2010–2019),” Medical Mycology 62, no. 5 (2024): myae048, 10.1093/mmy/myae048. 38684477

[18] J. I. Bittencourt , R. M. de Oliveira , and Z. F. Coutinho , “Paracoccidioidomycosis Mortality in the State of Paraná, Brazil, 1980/1998,” Cadernos de Saúde Pública 21, no. 6 (2005): 1856–1864, 10.1590/s0102-311x2005000600035. 16410872

[19] M. C. Gadêlha , G. C. W. Leandro , D. G. Ferreira , et al., “Spatiotemporal Patterns of Acute Paracoccidioidomycosis Hospitalizations in Brazil, 2014–2023,” Revista do Instituto de Medicina Tropical de São Paulo 67 (2025): e81, 10.1590/S1678-9946202567081. 41221949 PMC12600024

[20] I. Spinelli , L. R. de Macedo , and G. F. Ferreira , “Paracoccidioidomycosis‐Related Hospitalization and Mortality Rates in the State of São Paulo, Brazil: A Study Using Socioeconomic, Demographic, and Climatic Predictors,” Medical Mycology 63, no. 2 (2025): myaf014, 10.1093/mmy/myaf014. 39914446

[21] Z. F. Coutinho , B. Wanke , C. Travassos , R. M. Oliveira , D. R. Xavier , and C. E. Coimbra, Jr. , “Hospital Morbidity due to Paracoccidioidomycosis in Brazil (1998–2006),” Tropical Medicine & International Health 20, no. 5 (2015): 673–680, 10.1111/tmi.12472. 25645820

[22] Z. F. Coutinho , D. Silva , M. Lazera , et al., “Paracoccidioidomycosis Mortality in Brazil (1980–1995),” Cadernos de Saúde Pública 18, no. 5 (2002): 1441–1454, 10.1590/s0102-311x2002000500037. 12244377

[23] M. Prado , M. B. Silva , R. Laurenti , L. R. Travassos , and C. P. Taborda , “Mortality due to Systemic Mycoses as a Primary Cause of Death or in Association With AIDS in Brazil: A Review From 1996 to 2006,” Memórias do Instituto Oswaldo Cruz 104, no. 3 (2009): 513–521, 10.1590/s0074-0276200900030001. 19547881

[24] I. M. S. Suguiura and M. A. Ono , “Compulsory Notification of Paracoccidioidomycosis: A 14‐Year Retrospective Study of the Disease in the State of Paraná, Brazil,” Mycoses 65, no. 3 (2022): 354–361, 10.1111/myc.13417. 34936142

[25] M. Nakamura‐Pereira , W. Mendes‐Silva , M. A. Dias , M. E. Reichenheim , and G. Lobato , “The Hospital Information System of the Brazilian Unified National Health System: A Performance Evaluation for Auditing Maternal Near Miss,” Cadernos de Saúde Pública 29, no. 7 (2013): 1333–1345, 10.1590/s0102-311x2013000700008. 23843001

[26] Brasil , Sistema de Informação Sobre Mortalidade (SIM) (Secr. Vigilância em Saúde—Dep. Análise Epidemiológica e Vigilância Doenças Não Transm, 2025), https://www.gov.br/saude/pt‐br/composicao/svsa/sistemas‐de‐informacao/sim.

[27] Brasil , SIH—Sistema de Informação Hospitalar do SUS: Manual Técnico Operacional do Sistema. Secr. Atenção à Saúde (Secretaria de Atenção à Saúde, Departamento de Regulação, Avaliação e Controle/Coordenação Geral de Sistemas de Informação, 2017), 1–103, http://www2.datasus.gov.br/SIHD/manuais.

[28] IBGE , “Panorama do Censo 2022. Inst. Bras. Geogr. e Estatística,” 2025, https://censo2022.ibge.gov.br/panorama/?localidade=BR.

[29] Brasil , “Cadastro Nacional de Estabelecimentos de Saúde. Ministério da Saúde—Cadastro Nac. Estabel. Saúde,” 2025, http://cnes.datasus.gov.br/.

[30] IBGE , Classificação e caracterização dos espaços rurais e urbanos do Brasil: uma primeira aproximação, 1st ed., ed. Coordenação de Geografia (Estudos e Pesquisas, 2017), IBGE, https://biblioteca.ibge.gov.br/index.php/biblioteca‐catalogo?view=detalhes&id=2100643.

[31] Mapbiomas , Destaques agropecuária no Brasil (1985–2022) (Mapbiomas, 2022), https://brasil.mapbiomas.org/wp‐content/uploads/sites/4/2023/10/FACT_MapBiomas_Agropecuaria_04.10_v2.pdf.

[32] Brasil , Transferência de Arquivos – DATASUS (Ministério da Saúde, Dep. Informação e Informática do Sist. Único Saúde, 2025), https://datasus.saude.gov.br/transferencia‐de‐arquivos/.

[33] WHO , “International Statistical Classification of Diseases and Related Health Problems 10th Revision. ICD‐10 Version 2019,” 2025, https://icd.who.int/browse10/2019/en.

[34] Brasil , População Residente—Estudo de Estimativas Populacionais por Município, Idade e Sexo 2000–2024 ‐ Brasil (Dep. Informação e Informática do Sist. Único Saúde é o Dep. informática do Sist. Único Saúde do Bras, 2025), http://tabnet.datasus.gov.br/cgi/deftohtm.exe?ibge/cnv/popsvs2024br.def.

[35] Brasil , Tabwin—Tabulador de dados para Windows (Dep. Informação e Informática do Sist. Único Saúde, 2025), ftp://ftp.datasus.gov.br/tabwin/tabwin/TAB415.zip.

[36] CIDACS , Índice Brasileiro de Privação (IBP) (Cent. Integr. Dados e Conhecimentos para Saúde, Inst. Gonçalo Moniz—Fundação Oswaldo Cruz no estado da Bahia, 2025), https://cidacs.bahia.fiocruz.br/ibp/.

[37] M. Y. T. Ichihara , D. Ramos , P. Rebouças , et al., “Area Deprivation Measures Used in Brazil: A Scoping Review,” Revista de Saúde Pública 52 (2018): 83, 10.11606/S1518-8787.2018052000933. 30183845 PMC6122878

[38] H. J. Kim , M. P. Fay , E. J. Feuer , and D. N. Midthune , “Permutation Tests for Joinpoint Regression With Applications to Cancer Rates,” Statistics in Medicine 19, no. 3 (2000): 335–351, 10.1002/(sici)1097-0258(20000215)19:3<335::aid-sim336>3.0.co;2-z. 10649300

[39] G. F. Jenks , The Data Model Concept in Statistical Mapping, 1967 International Yearbook of Cartography (C. Vertelsmans Verlag, 1967), 186–190.

[40] R. Martinez , “Paracoccidioidomycosis: The Dimension of the Problem of a Neglected Disease,” Revista da Sociedade Brasileira de Medicina Tropical 43, no. 4 (2010): 480, 10.1590/s0037-86822010000400034. 20802960

[41] E. A. Souza , M. M. Cruz , A. F. Ferreira , et al., “Hospital Case Fatality and Mortality Related to Chagas Disease in Brazil Over Two Decades,” BMC Public Health 24, no. 1 (2024): 2282, 10.1186/s12889-024-19618-z. 39174935 PMC11342737

[42] J. Paim , C. Travassos , C. Almeida , L. Bahia , and J. Macinko , “The Brazilian Health System: History, Advances, and Challenges,” Lancet 377, no. 9779 (2011): 1778–1797, 10.1016/S0140-6736(11)60054-8. 21561655

[43] A. H. Santo , “Paracoccidioidomycosis‐Related Mortality Trend, State of São Paulo, Brazil: A Study Using Multiple Causes of Death,” Revista Panamericana de Salud Pública 23, no. 5 (2008): 313–324. 18510791 10.1590/s1020-49892008000500003

[44] WHO , “Neglected Tropical Diseases: Progress Towards Recovery From COVID‐19—2022 Update,” Weekly Epidemiological Record 97, no. 38 (2022): 465–480, https://iris.who.int/bitstream/handle/10665/363108/WER9738‐eng‐fre.pdf?sequence=1.

[45] IBGE , Brasil tem 1,7 milhão de indígenas e mais da metade deles vive na Amazônia Legal (Agência IBGE Notícias—Censo 2022, 2023), https://agenciadenoticias.ibge.gov.br/agencia‐noticias/2012‐agencia‐de‐noticias/noticias/37565‐brasil‐tem‐1‐7‐milhao‐de‐indigenas‐e‐mais‐da‐metade‐deles‐vive‐na‐amazonia‐legal.

[46] L. V. Bernardelli , G. H. L. Castro , J. R. Gobi , E. Michellon , and J. E. R. V. Filho , Formalidade do mercado de trabalho e produção agrícola no Brasil (Texto para discussão / Instituto de Pesquisa Econômica Aplicada, 2020), https://repositorio.ipea.gov.br/server/api/core/bitstreams/dedfb4fd‐35cf‐4802‐bcdb‐313096a730ff/content.

[47] E. M. Falcão , P. M. de Macedo , D. F. S. Freitas , et al., “Paracoccidioidomycosis in People Living With HIV/AIDS: A Historical Retrospective Cohort Study in a National Reference Center for Infectious Diseases, Rio de Janeiro, Brazil,” PLoS Neglected Tropical Diseases 16, no. 6 (2022): e0010529, 10.1371/journal.pntd.0010529. 35704666 PMC9239448

[48] F. A. Almeida , F. F. Neves , D. J. Mora , et al., “Paracoccidioidomycosis in Brazilian Patients With and Without Human Immunodeficiency Virus Infection,” American Journal of Tropical Medicine and Hygiene 96, no. 2 (2017): 368–372, 10.4269/ajtmh.16-0254. 27895278 PMC5303038

[49] P. M. Macedo , R. Almeida‐Paes , M. A. Almeida , et al., “Paracoccidioidomycosis due to *Paracoccidioides brasiliensis* S1 Plus HIV Co‐Infection,” Memórias do Instituto Oswaldo Cruz 113, no. 3 (2018): 167–172, 10.1590/0074-02760170310. 29412355 PMC5804308

[50] A. F. Ferreira , E. A. de Souza , M. D. S. Lima , et al., “Mortality From Leprosy in Highly Endemic Contexts: Integrated Temporal‐Spatial Analysis in Brazil,” Revista Panamericana de Salud Publica/Pan American Journal of Public Health 43 (2019): e87, 10.26633/RPSP.2019.87. 31768181 PMC6830300

[51] Brasil , Portaria n° 264, de 17 de fevereiro de 2020. Altera a Portaria de Consolidação n° 4/GM/MS, de 28 de setembro de 2017 (Ministério da Saúde ‐ Gabinete do Ministro, 2020), https://bvsms.saude.gov.br/bvs/saudelegis/gm/2020/prt0264_19_02_2020.html.

[52] Brasil , Portaria n° 1.061, de 18 de maio de 2020. Revoga a Portaria n° 264, de 17 de fevereiro de 2020, e altera a Portaria de Consolidação n° 4/GM/MS, de 28 de setembro de 2017 (Ministério da Saúde ‐ Gabinete do Ministro, 2020), https://bvsms.saude.gov.br/bvs/saudelegis/gm/2020/prt1061_29_05_2020.html.

[53] Brasil , Nota Informativa n^o^ 9/2023‐CGTM/DATHI/SVSA/MS (Departamento de HIV, Aids, Tuberculose, Hepatites Virais e Infecções Sexualmente Transmissíveis, 2023), https://www.gov.br/aids/pt‐br/central‐de‐conteudo/notas‐informativas/2023/sei_ms‐0033993525‐nota‐informativa.pdf/@@download/file.

[54] A. H. Santo , “Epidemiological Potential of Multiple‐Cause‐Of‐Death Data Listed on Death Certificates, Brazil, 2003,” Revista Panamericana de Salud Pública 22, no. 3 (2007): 178–186, 10.1590/s1020-49892007000800004. 18062852

[55] C. Piffaretti , M. Moreno‐Betancur , A. Lamarche‐Vadel , and G. Rey , “Quantifying Cause‐Related Mortality by Weighting Multiple Causes of Death,” Bulletin of the World Health Organization 94, no. 12 (2016): 870–879, 10.2471/BLT.16.172189. 27994280 PMC5153928

