# Supplementary material for: Paracoccidioidomycosis in Brazil: 25‐Year Nationwide Trends in Mortality, Hospitalisations and In‐Hospital Deaths of a Neglected Systemic Mycosis
Source: Trop Med Int Health. 2026 Mar 24;31(7):875–94. doi: 10.1111/tmi.70135 (PMC13331544; doi:10.1111/tmi.70135)
Supplement: Supplementary file 1 — Table S1: Mentions of paracoccidioidomycosis (B41) or the 10 leading ICD‐10 codes as underlying/primary or associated/secondary causes of mortality (SIM), hospital admissions and in‐hospital mortality (SIH‐SUS), Brazil, 2000–2024. [file TMI-31-875-s001.docx]

**Supplementary material – Table 1.** Mentions of paracoccidioidomycosis (B41) or the 10 leading ICD-10 codes as underlying/primary or associated/secondary causes of mortality (SIM), hospital admissions and in-hospital mortality (SIH-SUS), Brazil, 2000–2024.

| **ICD-10 description** | **Underlying cause/ Primary cause** | **Associated cause/ Secondary cause** | **Total** |
| --- | --- | --- | --- |
|  | **N (%)** | **N (%)** | **N (%)** |
| **Deaths** |  |  |  |
| **Total citations** | **2,980 (37.5)** | **4,971 (62.5)** | **7,951 (100.0)** |
| B40 Blastomycosis | 0 (0.0) | 6 (0.1) | 6 (0.1) |
| B40.0 Acute pulmonary blastomycosis | 54 (1.8) | 49 (1) | 103 (1.3) |
| B40.1 Chronic pulmonary blastomycosis | 45 (1.5) | 86 (1.7) | 131 (1.6) |
| B40.2 Pulmonary blastomycosis (unspecified) | 428 (14.4) | 678 (13.6) | 1,106 (13.9) |
| B40.3 Cutaneous blastomycosis | 10 (0.3) | 22 (0.4) | 32 (0.4) |
| B40.7 Disseminated blastomycosis | 130 (4.4) | 61 (1.2) | 191 (2.4) |
| B40.8 Other forms of blastomycosis | 30 (1) | 65 (1.3) | 95 (1.2) |
| B40.9 Blastomycosis (unspecified) | 326 (10.9) | 782 (15.7) | 1,108 (13.9) |
| B41 Paracoccidioidomycosis | 0 (0.0) | 5 (0.1) | 5 (0.1) |
| B41.0 Pulmonary paracoccidioidomycosis | 738 (24.8) | 989 (19.9) | 1,727 (21.7) |
| B41.7 Disseminated paracoccidioidomycosis | 460 (15.4) | 281 (5.7) | 741 (9.3) |
| B41.8 Other forms of paracoccidioidomycosis | 102 (3.4) | 167 (3.4) | 269 (3.4) |
| B41.9 Paracoccidioidomycosis (unspecified) | 657 (22.0) | 1,780 (35.8) | 2,437 (30.7) |
| **Top 10 ICD-10** |  |  |  |
| **Total citations** | **1,924 (13.0)** | **12,834 (87.0)** | **14,758 (100.0)** |
| A41.9 Septicaemia (Unspecified) | 7 (0.4) | 1,347 (10.5) | 1,354 (9.2) |
| J96.9 Respiratory failure (Unspecified) | 4 (0.2) | 1,076 (8.4) | 1,080 (7.3) |
| J44.9 Chronic obstructive pulmonary disease (Unspecified) | 172 (8.9) | 717 (5.6) | 889 (6.0) |
| J96.0 Acute respiratory failure | 1 (0.1) | 857 (6.7) | 858 (5.8) |
| J18.9 Pneumonia (Unspecified) | 18 (0.9) | 741 (5.8) | 759 (5.1) |
| R68.8 Other specified general symptoms and signs | 0 (0.0) | 440 (3.4) | 440 (3.0) |
| R09.2 Respiratory arrest | 0 (0.0) | 436 (3.4) | 436 (3.0) |
| A16.2 Tuberculosis of the respiratory tract, without bacteriological or histological confirmation | 109 (5.7) | 157 (1.2) | 266 (1.8) |
| F17.2 Dependence syndrome | 10 (0.5) | 241 (1.9) | 251 (1.7) |
| N17.9 Acute renal failure (Unspecified) | 27 (1.4) | 219 (1.7) | 246 (1.7) |
| **Hospitalisations** |  |  |  |
| **Total citations** | **17,056 (96.4)** | **645 (3.6)** | **17,701 (100.0)** |
| B40 Blastomycosis | 12 (0.1) | 9 (1.4) | 21 (0.1) |
| B40.0 Acute pulmonary blastomycosis | 1,307 (7.7) | 14 (2.2) | 1,321 (7.5) |
| B40.1 Chronic pulmonary blastomycosis | 615 (3.6) | 7 (1.1) | 622 (3.5) |
| B40.2 Pulmonary blastomycosis (Unspecified) | 3,195 (18.7) | 32 (5) | 3,227 (18.2) |
| B40.3 Cutaneous blastomycosis | 212 (1.2) | 2 (0.3) | 214 (1.2) |
| B40.7 Disseminated blastomycosis | 376 (2.2) | 8 (1.2) | 384 (2.2) |
| B40.8 Other forms of blastomycosis | 916 (5.4) | 16 (2.5) | 932 (5.3) |
| B40.9 Blastomycosis (Unspecified) | 2,284 (13.4) | 36 (5.6) | 2,320 (13.1) |
| B41 Paracoccidioidomycosis | 63 (0.4) | 31 (4.8) | 94 (0.5) |
| B41.0 Pulmonary paracoccidioidomycosis | 2,288 (13.4) | 131 (20.3) | 2,419 (13.7) |
| B41.7 Disseminated paracoccidioidomycosis | 1521 (8.9) | 118 (18.3) | 1,639 (9.3) |
| B41.8 Other forms of paracoccidioidomycosis | 1022 (6) | 75 (11.6) | 1,097 (6.2) |
| B41.9 Paracoccidioidomycosis (Unspecified) | 3245 (19) | 166 (25.7) | 3,411 (19.3) |
| **Top 10 ICD-10** |  |  |  |
| **Total citations** | **1,183 (10.3)** | **10,311 (89.7)** | **11,494 (100.0)** |
| B57.2 Chagas disease (chronic) with cardiac involvement | 0 (0) | 3,513 (34.1) | 3,513 (30.6) |
| B57.3 Chagas disease (chronic) with digestive tract involvement | 0 (0) | 1,512 (14.7) | 1,512 (13.2) |
| B57.0 Acute form of Chagas disease with cardiac involvement | 0 (0) | 575 (5.6) | 575 (5.0) |
| I98.1 Cardiovascular disorders in other infectious and parasitic diseases classified elsewhere | 0 (0) | 406 (3.9) | 406 (3.5) |
| B57.5 Chagas disease (chronic) with involvement of other organs | 0 (0) | 314 (3) | 314 (2.7) |
| I41.2 Myocarditis in other infectious and parasitic diseases classified elsewhere | 0 (0) | 275 (2.7) | 275 (2.4) |
| K23.1 Megaesophagus in Chagas disease | 0 (0) | 274 (2.7) | 274 (2.4) |
| K93.1 Megacolon in Chagas disease | 0 (0) | 232 (2.3) | 232 (2.0) |
| B57.1 Acute form of Chagas disease, without cardiac involvement | 0 (0) | 174 (1.7) | 174 (1.5) |
| I10 Essential hypertension | 4 (0.3) | 122 (1.2) | 126 (1.1) |
| **Hospital mortality** |  |  |  |
| **Total citations** | **962 (88.9)** | **120 (11.1)** | **1,082 (100.0)** |
| B40 Blastomycosis | 0 (0.0) | 4 (3.3) | 4 (0.4) |
| B40.0 Acute pulmonary blastomycosis | 121 (12.6) | 4 (3.3) | 125 (11.6) |
| B40.1 Chronic pulmonary blastomycosis | 58 (6.0) | 2 (1.7) | 60 (5.5) |
| B40.2 Pulmonary blastomycosis (Unspecified) | 229 (23.8) | 7 (5.8) | 236 (21.8) |
| B40.3 Cutaneous blastomycosis | 2 (0.2) | 0 (0.0) | 2 (0.2) |
| B40.7 Disseminated blastomycosis | 17 (1.8) | 3 (2.5) | 20 (1.8) |
| B40.8 Other forms of blastomycosis | 19 (2.0) | 3 (2.5) | 22 (2.0) |
| B40.9 Blastomycosis (unspecified) | 114 (11.9) | 6 (5.0) | 120 (11.1) |
| B41 Paracoccidioidomycosis | 3 (0.3) | 3 (2.5) | 6 (0.6) |
| B41.0 Pulmonary paracoccidioidomycosis | 132 (13.7) | 30 (25.0) | 162 (15.0) |
| B41.7 Disseminated paracoccidioidomycosis | 70 (7.3) | 27 (22.5) | 97 (9.0) |
| B41.8 Other forms of paracoccidioidomycosis | 31 (3.2) | 5 (4.2) | 36 (3.3) |
| B41.9 Paracoccidioidomycosis (Unspecified) | 166 (17.3) | 26 (21.7) | 192 (17.7) |
| **Top 10 ICD-10** |  |  |  |
| **Total citations** | **174 (12.8)** | **1,182 (87.2)** | **1,356 (100.0)** |
| B57.2 Chagas disease (chronic) with cardiac involvement | 0 (0.0) | 435 (36.8) | 435 (32.1) |
| B57.3 Chagas disease (chronic) with digestive tract involvement | 0 (0.0) | 84 (7.1) | 84 (6.2) |
| A41.9 Septicaemia (Unspecified) | 39 (22.4) | 22 (1.9) | 61 (4.5) |
| B57.0 Acute form of Chagas disease with cardiac involvement | 0 (0.0) | 56 (4.7) | 56 (4.1) |
| I98.1 Cardiovascular disorders in other infectious and parasitic diseases classified elsewhere | 0 (0.0) | 54 (4.6) | 54 (4.0) |
| B57.5 Chagas disease (chronic) with involvement of other organs | 0 (0.0) | 39 (3.3) | 39 (2.9) |
| J96.0 Acute respiratory failure | 15 (8.6) | 23 (1.9) | 38 (2.8) |
| R09.2 Respiratory arrest | 0 (0.0) | 29 (2.5) | 29 (2.1) |
| N17.9 Acute renal failure (Unspecified) | 0 (0.0) | 26 (2.2) | 26 (1.9) |
| B57.1 Acute form of Chagas disease, without cardiac involvement | 0 (0.0) | 23 (1.9) | 23 (1.7) |
